# Social support and cognition in a racially and ethnically diverse cohort: the role of neighborhood disadvantage

**DOI:** 10.3389/fneur.2026.1918066

**Published:** 2026-10-13

**Authors:** Tasha Rhoads, Alec C. Neale, Kimberly Cobos, Katie Stypulkowski, Christina G. Wong, Sid E. O’Bryant, Amy J. H. Kind, Justin B. Miller

**Affiliations:** 1 Department of Neurology, University of Washington School of Medicine, Seattle, WA, United States; 2 Department of Neurology, Yale University School of Medicine, New Haven, CT, United States; 3 Lou Ruvo Center for Brain Health, Cleveland Clinic, Las Vegas, NV, United States; 4 Department of Neurology, Westchester Medical Center, Valhalla, NY, United States; 5 Institute for Translational Research, University of North Texas Health Science Center, Fort Worth, TX, United States; 6 Center for Health Disparities Research, University of Wisconsin School of Medicine and Public Health, Madison, WI, United States; 7 Department of Medicine, Division of Geriatrics and Gerontology, University of Wisconsin School of Medicine and Public Health, Madison, WI, United States

**Keywords:** aging, brain health, cognition, neighborhood disadvantage, social support

## Abstract

**Background:**

Social support is positively associated with cognitive health, but which dimensions of support matter most and how this relationship varies across environmental contexts remains under-explored. This study examined whether neighborhood disadvantage, measured by the Area Deprivation Index (ADI), moderates the relationship between social support and cognition in an ethnocracially diverse cohort of adults aged 50 and older.

**Methods:**

Data were analyzed from the Health and Aging Brain Study-Health Disparities (HABS-HD), which used community-based participatory research methods to recruit Non-Hispanic Black (NHB), Hispanic, and Non-Hispanic White (NHW) participants across the cognitive spectrum (normal, mild cognitive impairment, dementia). Neighborhood disadvantage was dichotomized as low versus high, and three Interpersonal Support Evaluation subscales (Tangible, Companion, and Emotional Support) were assessed. For each racial/ethnic group, a social support × ADI interaction term was tested as the primary analysis. Pooled and ADI-stratified associations between each subscale and cognition (Mini-Mental State Exam; MMSE) were examined as secondary, exploratory analyses.

**Results:**

The interaction test did not provide evidence that neighborhood disadvantage moderates the association between social support and cognition in any racial/ethnic group (all *p* > 0.23). Independent of neighborhood disadvantage, Tangible Support was significantly and positively associated with cognition across all three groups, while Emotional Support was significantly associated with cognition among NHB and Hispanic individuals. In exploratory subgroup analyses stratified by neighborhood disadvantage, Tangible and Emotional Support were significant among NHB and Hispanic individuals in lower-disadvantage areas (ADI 1–4), and Tangible Support among NHW individuals in this stratum. No subscale predicted cognition in the highest-disadvantage areas.

**Conclusion:**

Tangible Support, and to a lesser extent Emotional Support, showed positive associations with cognition across ethnoracial groups, with the most consistent associations observed among NHB and Hispanic individuals in lower-disadvantage neighborhoods, though these associations attenuated in several sensitivity analyses. In exploratory subgroup analyses, significant associations were not observed among individuals residing in the most disadvantaged neighborhoods, though these findings should be interpreted cautiously given unequal strata sample sizes and warrant confirmation in future studies.

## 1 Background

Population estimates indicate that over 6 million older adults in the United States currently live with Alzheimer’s disease (AD). As the “baby boom” generation ages, this number is projected to increase to 13.8 million by 2060 (1). Although significant strides have been made in AD treatments to slow disease progression, pharmacological agents that reverse the disease process are not currently available (2). Therefore, understanding modifiable risk and protective factors for dementia is crucial for preventing and ameliorating the effects of cognitive decline. The 2024 report from the Lancet Commission on Dementia Prevention, Intervention, and Care identified 14 modifiable risk factors for dementia across the life course (3). Many of the identified risk factors, such as depression and social isolation, have been shown to be elevated in lower socioeconomic status (SES) groups (4, 5). As it stands, both neighborhood deprivation and race/ethnicity are inextricably linked with SES and health disparities through entrenched structural and health pathways (6, 7). Further, both neighborhood disadvantage and racial/ethnic identity are tied to increased dementia risk, with evidence of both shared and unique contributions (8). As such, reducing risk of dementia is particularly important among racially and ethnically diverse groups, given observed ethnoracial disparities in rates of AD and related dementias [ADRDs] (9, 10). For example, African American older adults are twice as likely to be diagnosed with ADRD while Hispanic older adults are 1.5 times as likely compared to NHW older adults (1, 11).

Among the variety of modifiable risk factors that may influence cognitive decline, social isolation represents a late life risk factor for dementia, while social support has emerged as one factor that consistently shows a positive and robust association with cognition (3, 12–16). Although related, social isolation and social support are conceptually distinct constructs. Social isolation refers to the objective absence or infrequency of social contact, while social support reflects the perceived or actual availability of assistance within existing relationships (17). This divergence may be particularly pronounced in under-resourced neighborhoods, where structural barriers can limit the translation of social contact into perceived or received support.

Social support has been defined as the available support for an individual through social ties with other people, groups, or the community and may take on various forms including virtual, perceived, real, transitory, or continuous (18). Specifically, meta-analytic evidence provides convincing support to suggest that social relations serve to build cognitive reserve and promote healthy cognitive aging (19–21). As such, older adults with stronger social networks and greater perceived social support tend to perform better on cognitive tests (14, 22). Prior literature has also suggested that strong social connections can provide feelings of belonging, purpose, and security, which positively impact emotional well-being and indirectly benefit cognitive function (23). Social support in the form of supportive listening may specifically increase cognitive resilience, as it has been shown to independently modify the association between lower total cerebral volume and poorer cognitive function that would otherwise indicate increased risk for ADRDs at the preclinical stage (24). However, as this literature is largely cross-sectional, reverse causation (i.e., better cognitive functioning enabling the maintenance of social relationships) cannot be ruled out. These findings also align with complimentary theoretical explanations that help frame such phenomena as facets of cognitive resilience such that social support may yield a buffering effect, reducing the impact of daily stressors and adverse psychological and health outcomes (25).

Previous research suggests that the relationship between social support and cognition differs as a function of race and cultural background. For example, Hamlin et al. (2022) found that older Black adults participate in social activities less frequently than non-Hispanic White adults while Katz et al. (2020) found that number of close family members showed strong positive associations with executive functioning performance among ethnic and racial minority groups (26, 27). Culture also appears to play a role in shaping the type of social support that matters most. For example, a study by Zamora-Macorra et al. (2017) found that emotional support from family members was particularly beneficial for cognition among Mexican adults, likely reflecting core cultural values often placed on close, supportive family ties within Hispanic communities (28). Conversely, Yeh and Liu (2003) showed that instrumental support, such as practical help with daily tasks, was more important for cognitive function in older Taiwanese adults, a finding that may reflect a strategic emphasis on communal care over the potential social costs of explicit emotional disclosure (29).

Despite these findings, a critical gap remains in how factors such as socioeconomic status or neighborhood context may impact the association between social support and cognition. Although one study found that social support and loneliness varies among older adults based on neighborhood type (e.g., cities, towns, rural areas), research examining the link between social support and neighborhood characteristics is sparse (30). The purpose of the current study was to examine associations between various types of social support and cognitive functioning and to evaluate whether these associations differed according to neighborhood disadvantage, as measured by the Area Deprivation Index (ADI), in a diverse sample of adults and older adults with and without cognitive impairment. Few studies have examined whether associations between social resources and cognition differ across levels of neighborhood disadvantage, although preliminary findings indicate that relationship quality moderates the association between neighborhood disadvantage and cognition among certain older adults, specifically highlighting that support from relatives is linked to better cognitive outcomes for Hispanic and Black, but not White individuals (31, 32). Building upon these frameworks, we hypothesized that neighborhood disadvantage would moderate the association between social support and cognition, such that this association would be stronger for those living in less disadvantaged neighborhoods (Hypothesis 1). We tested this hypothesis formally via a social support x ADI interaction term, with stratified subgroup comparisons serving as a secondary, exploratory component to that test. We further hypothesized that emotional support would serve as a key factor in the relationship between social support and cognition, reflecting its established importance in cognitive health based on prior systematic reviews (Hypothesis 2) (12). The goal of this research is to investigate the impact of individual and contextual factors on the relationship between social support and cognitive functioning. By clarifying how social support and neighborhood context relate to cognition, these findings may help inform future clinical recommendations and intervention research.

## 2 Methods

### 2.1 Participants

Data included in this cross-sectional study were obtained from the Health and Aging Brain Study – Health Disparities (HABS-HD) study, an ongoing observational study enrolling community-dwelling adults and older adults from the Dallas/Fort Worth metropolitan area of Texas to study health disparities in cognitive aging among Hispanic (majority Mexican American), non-Hispanic White (NHW), and non-Hispanic Black (NHB) individuals (33). HABS-HD methods have been published elsewhere (33, 34). In brief, participation (in either Spanish or English) involves interviews with both participant and informant, a clinical exam, completion of self-report measures, neuropsychological testing, neuroimaging, and blood draw for clinical labs and biomarker characterization every two years. All participants received a research diagnosis determined by consensus conference of cognitively normal (CN), mild cognitive impairment (MCI), or dementia. Baseline data drawn from all three racial/ethnic cohorts who participated in the study between 2017 and 2023 were included in this project. Exclusion criteria included: (1) lack of available ADI data and (2) age less than 50. The HABS-HD study is conducted under IRB-approved protocols at the University of North Texas Health Sciences Center and each participant (or their legal representative) provided written informed consent.

### 2.2 Measures

#### 2.2.1 Mini-Mental State Exam (MMSE)

The MMSE is a simple pen-and-paper cognitive screening measure that includes tests of orientation, concentration, attention, verbal memory, naming, and visuospatial skills. The Total Score (range: 0–30) was used in analyses (35).

#### 2.2.2 Interpersonal Support Evaluation List – Short Version

A 12-item questionnaire was used to assess perceptions of social support for individual participants. This self-report questionnaire contains three subscales designed to measure three different dimensions of perceived social support: (1) Companion Support (e.g., having support from companions or someone to accompany an individual during activities), (2) Emotional [or Appraisal] Support (e.g., perceiving that one has others to confide in or turn to for advice), and (3) Tangible Support (e.g., perceiving that one has access to assistance with household tasks). Each dimension is measured by 4 items on a 4-point scale ranging from “Definitely True” to “Definitely False” with reverse scoring implemented for certain items (higher total scores correspond to greater levels of support). Items comprising the Interpersonal Support Evaluation List with embedded subscales are reproduced in the Appendix (36).

#### 2.2.3 Area Deprivation Index (ADI)

The ADI is a well-validated and widely utilized measure of neighborhood disadvantage that has been used across a variety of clinical groups, including older adults and ethnoracially diverse populations (37–41). Methods to describe its development and implementation are reported elsewhere, and the resulting Neighborhood Atlas has been made freely available (42–44). Briefly, the ADI offers a quantifiable rating of neighborhood disadvantage by weighting and aggregating relevant factors (e.g., income, education, employment, and housing quality) for a given Census block, which is geocoded based on current residential address. In this study, Texas state ADI decile rankings were collapsed into quintiles to reduce the number of required comparisons, with Quintile 1 representing the least disadvantaged areas (i.e., 20% least disadvantaged neighborhoods) and Quintile 5 reflecting the areas of the greatest disadvantage (i.e., 20% most disadvantaged neighborhoods). The overall sample was dichotomized as the top 20% (highest level of deprivation; ADI Quintile 5) and the bottom 80% of neighborhood disadvantage (lower levels of deprivation; ADI Quintiles 1–4), according to the most common dichotomization method used in studies examining ADI (45, 46). This method was selected given that prior work within our group using ADI quintiles has shown that the highest quintile (i.e., representing greatest levels of structural disadvantage) functions in a qualitatively distinct manner rather than simply capturing the upper end of a continuous gradient (47–49). As a robustness check, we also examined ADI as a continuous, quintile-based measure (see Sensitivity Analyses).

### 2.3 Statistical analyses

Demographic covariates included in primary analyses were chronological age (measured in years), highest educational attainment (quantified in years of formal schooling completed), biological sex (dichotomized as male or female), and neighborhood disadvantage, operationalized via the ADI (stratified into high versus low disadvantage levels). Continuous and categorical variables were compared across neighborhood disadvantage levels using *t*-tests or *χ^2^* tests, respectively. Preliminary boxplot visualization, implemented within each ethnoracial group independently following dichotomization of the sample by ADI classification, identified a small number of extreme values in the primary outcome (MMSE; defined as exceeding 1.5 times the interquartile range below the first quartile or above the third quartile). No participants were excluded from primary analyses on this basis; instead, the sensitivity of primary model estimates to potentially influential cases was evaluated formally using regression-diagnostic criteria (see Sensitivity Analyses, Section 3.4a). Given the exploratory nature of these analyses and the conceptual overlap among the moderately correlated social support subscales, we did not apply a Bonferroni correction for multiple comparisons to avoid unnecessarily inflating the risk of Type II errors and obscuring meaningful effects.

To formally evaluate whether neighborhood disadvantage moderates the association between social support and global cognition, we conducted hierarchical regression analyses within each racial/ethnic group, entering a social support × ADI interaction term after accounting for demographic covariates and the main effects of support and ADI. Across all nine models, three support subscales (Tangible, Emotional, and Companion support) evaluated within each of three racial/ethnic groups, the interaction term did not reach statistical significance (all *p* > 0.23; Table 1). The incremental variance explained by the interaction term was negligible in every model (ΔR^2^ ≤ 0.001). All models retain the full analytic sample with valid MMSE data.

**Table 1:** Support × ADI interaction and pooled main-effect regression models predicting global cognition (MMSE), by racial/ethnic group.

Race/ethnicity	Support subscale	*N*	Interaction ΔR^2^ (*p*)	Main effect B (SE)	*β*	*t*	*p*	VIF	Tolerance
Black	Tangible	831	ΔR^2^ = 0.001, *p* = 0.348	B = 0.221 (0.091)	0.082	2.44	0.015	1.07	0.938
Black	Emotional	830	ΔR^2^ < 0.001, *p* = 0.909	B = 0.178 (0.085)	0.069	2.09	0.037	1.11	0.900
Black	Companion	832	ΔR^2^ < 0.001, *p* = 0.591	B = 0.129 (0.087)	0.048	1.49	0.137	1.05	0.957
Hispanic	Tangible	1,167	ΔR^2^ < 0.001, *p* = 0.346	B = 0.301 (0.087)	0.085	3.45	< 0.001	1.04	0.962
Hispanic	Emotional	1,166	ΔR^2^ < 0.001, *p* = 0.657	B = 0.183 (0.087)	0.052	2.11	0.035	1.04	0.963
Hispanic	Companion	1,167	ΔR^2^ < 0.001, *p* = 0.550	B = 0.120 (0.090)	0.033	1.33	0.184	1.03	0.974
White	Tangible	1,154	ΔR^2^ < 0.001, *p* = 0.414	B = 0.250 (0.067)	0.106	3.75	< 0.001	1.01	0.986
White	Emotional	1,156	ΔR^2^ < 0.001, *p* = 0.694	B = 0.120 (0.067)	0.051	1.78	0.076	1.04	0.964
White	Companion	1,153	ΔR^2^ < 0.001, *p* = 0.230	B = 0.076 (0.061)	0.036	1.25	0.212	1.02	0.977

Each support subscale (Tangible, Emotional, Companion) was modeled independently in its own regression, adjusting for age, sex, education, and dichotomous ADI (top quintile vs. remaining quintiles). Interaction ΔR^2^ (*p*) reflects the change in R^2^ when the Support × ADI interaction term was added in a third step. *p < 0.05, **p < 0.01, ***p < 0.001.

Independent of the interaction analyses, the pooled association between support and cognition (i.e., collapsing across ADI level within each racial/ethnic group) was examined in Step 2 of each model. Tangible Support was significantly associated with cognition among Black (B = 0.221, SE = 0.091, *p* = 0.015), Hispanic (B = 0.301, SE = 0.087, *p* < 0.001), and White participants (B = 0.250, SE = 0.067, *p* < 0.001). Emotional Support was significantly associated with cognition among Black (B = 0.178, *p* = 0.037) and Hispanic participants (B = 0.183, *p* = 0.035), but not White participants (B = 0.120, *p* = 0.076). Companion Support was not significantly associated with cognition in any group (Black: B = 0.129, SE = 0.087, *p* = 0.137; Hispanic: B = 0.120, SE = 0.090, *p* = 0.184; White: B = 0.076, SE = 0.061, *p* = 0.212). Because nine comparisons were evaluated at this step, we note that only the Tangible Support associations among Hispanic and White participants would survive a Bonferroni correction (α = 0.0056). The remaining associations, while nominally significant, should be interpreted with appropriate caution given the number of comparisons conducted. Variance inflation factors (VIFs) for all predictors across these nine models ranged from 1.00–1.16 (tolerance = 0.86–0.996), indicating no meaningful multicollinearity (Table 1). The three support subscales were moderately intercorrelated (Tangible–Emotional r = 0.59, Tangible–Companion r = 0.58, Emotional–Companion r = 0.55, pooled across groups; correlations by racial/ethnic group ranged from 0.54–0.62), consistent with our decision to model each subscale in an independent regression rather than entering all three simultaneously. At the main-effect step (added after age, sex, education, and dichotomous ADI), the incremental variance explained by Tangible Support was small but statistically significant in all three groups (Black: ΔR^2^ = 0.006, *F*(1,825) = 5.95, *p* = 0.015; Hispanic: ΔR^2^ = 0.007, *F*(1,1,161) = 11.91, *p* < 0.001; White: ΔR^2^ = 0.011, *F*(1,1,148) = 14.06, *p* < 0.001), as was Emotional Support among Black (ΔR^2^ = 0.004, *F*(1,824) = 4.36, *p* = 0.037) and Hispanic participants (ΔR^2^ = 0.003, *F*(1,1,160) = 4.47, *p* = 0.035). The main-effect ΔR^2^ for Companion Support, and for Emotional Support among White participants, did not reach significance (all *p* ≥ 0.07).

As a secondary, exploratory complement to the primary interaction analysis, we also examined associations between social support and cognition within subgroups stratified by both dichotomized neighborhood disadvantage (ADI Quintiles 1–4 versus Quintile 5) and racial/ethnic group. In the first step of each hierarchical regression, baseline demographic factors (age, sex, and educational attainment) were entered to account for their established predictive variance on global cognitive scores. In the second step, each social support subscale was entered independently (i.e., in its own separate model, rather than simultaneously with the other two subscales), consistent with the approach used in the primary pooled analysis above and given that the three subscales are moderately intercorrelated. This stratified approach does not itself constitute a formal test of moderation and these subgroup comparisons should accordingly be interpreted as hypothesis-generating. Tests were 2-tailed, and statistical significance was set at *p* < 0.05 for all analyses. Data were analyzed using SPSS Version 23 (IBM).

To evaluate the robustness of these findings, we conducted four sensitivity analyses: (a) re-estimating models with MMSE outliers retained rather than excluded; (b) re-estimating models adjusting additionally for consensus cognitive diagnosis, depressive symptoms, and language of assessment; (c) restricting the analytic sample to cognitively normal participants only, to address the possibility of reverse causation; and (d) re-estimating the primary interaction and main-effect models using ADI as a continuous, quintile-based measure rather than a dichotomized one. Results of these analyses are reported in Section 3.4.

## 3 Results

Overall demographic characteristics stratified by neighborhood disadvantage level are presented in Table 2, including age, educational attainment, sex, social support subscales, and global cognition (MMSE).

**Table 2:** Demographic comparisons by neighborhood disadvantage level.

Variable	Low ND (ADI 1–4) *n* = 2,624	High ND (ADI 5) *n* = 548	*t* (d)	χ^2^ (V)
Age [*M (SD), Range*]	65.8 (8.6), 50–93	63.9 (8.4), 50–91	4.6*** (0.2)	–
Education [*M (SD), Range*]	14.1 (3.9)	10.4 (4.4)	19.4*** (0.9)	–
Sex [*n* (%)]			–	1.17 (0.02)
Female	1,619 (61.7%)	352 (64.2%)		
Male	1,006 (38.3%)	196 (35.8%)		
Race			–	268.8*** (0.29)
Black	652 (77.9%)	185 (22.1%)		
Hispanic	847 (72.1%)	327 (27.9%)		
White	1,125 (96.9%)	36 (3.1%)		
Cognitive diagnosis [*n* (%)]			–	23.1*** (0.09)
Cognitively normal	1,967 (75.0%)	356 (65.0%)		
Mild cognitive impairment	507 (19.3%)	148 (27.0%)		
Dementia	150 (5.7%)	44 (8.0%)		
Social support			–	
Companion	13.2 (2.6)	12.7 (2.7)	4.8*** (0.2)	–
Emotional	14.0 (2.5)	13.1 (2.6)	7.5*** (0.4)	–
Tangible	14.1 (2.2)	13.3 (2.4)	7.1*** (0.3)	–
Global cognition			–	
MMSE total	27.7 (3.0)	26.2 (3.6)	10.3*** (0.5)	–

d = Cohen’s d. V = Cramer’s V. ADI, Area Deprivation Index; ND, Neighborhood disadvantage; ADI Quintile 1, Least Disadvantaged; ADI Quintile 5, Most Disadvantaged; CN, Cognitively Normal; MCI, Mild Cognitive Impairment. ***p < 0.001.

After applying the exclusion criteria described above (lack of available ADI data and age under 50), the final analytic sample comprised 3,172 participants: 2,624 residing in areas of lower neighborhood disadvantage (ADI Quintiles 1–4) and 548 residing in areas of higher neighborhood disadvantage (ADI Quintile 5). This reflects inclusion of participants across the full cognitive spectrum (CN, MCI, and dementia. No participants were excluded from the primary analyses on the basis of an outlying MMSE score (see Sensitivity Analyses, Section 3.4a). Individuals living in the most disadvantaged areas had significantly fewer years of formal education, lower performance on a measure of global cognition, and less self-reported social support across all three subscales. Additionally, the high neighborhood disadvantage group contained a smaller proportion of White individuals relative to lower neighborhood disadvantage quintiles, was younger overall, and included a somewhat greater proportion of participants with MCI or dementia (35.0% vs. 25.0% in the lower-disadvantage group; χ^2^(2) = 23.12, *p* < 0.001, V = 0.09). Although these group differences were statistically significant, effect sizes and overall score distributions (i.e., MMSE scores and social support ratings were generally negatively skewed with a preponderance of values falling in higher ranges) importantly do not suggest that the present findings were confounded by demographic factors or psychometric artifact.

The formal test of our primary hypothesis (social support × ADI interaction) did not provide statistical evidence that neighborhood disadvantage moderates the association between social support and cognition in any racial/ethnic group. Therefore, the exploratory subgroup analyses reported in Section 3.3 should accordingly be interpreted as hypothesis-generating rather than confirmatory of Hypothesis 1.

### 3.1 Support × ADI interaction analyses (primary analysis)

Across all nine models (three support subscales × three racial/ethnic groups), the social support × ADI interaction term did not reach statistical significance (all *p* > 0.23), and the incremental variance explained by the interaction term was negligible in every model (ΔR^2^ ≤ 0.001; Table 1). These results do not provide statistical evidence that neighborhood disadvantage moderates the association between social support and cognition within any racial/ethnic group in this sample.

### 3.2 Pooled associations between social support and cognition

Table 1 presents the pooled (ADI-adjusted, not ADI-stratified) main-effect models for all nine subscale × racial/ethnic group combinations. Tangible Support was significantly associated with cognition among Black (B = 0.221, SE = 0.091, *p* = 0.015), Hispanic (B = 0.301, SE = 0.087, *p* < 0.001), and White participants (B = 0.250, SE = 0.067, *p* < 0.001). Emotional Support was significantly associated with cognition among Black (B = 0.178, SE = 0.085, *p* = 0.037) and Hispanic participants (B = 0.183, SE = 0.087, *p* = 0.035), but not White participants (B = 0.120, SE = 0.067, *p* = 0.076). Companion Support was not significantly associated with cognition in any group (Black: B = 0.129, *p* = 0.137; Hispanic: B = 0.120, *p* = 0.184; White: B = 0.076, *p* = 0.212). Variance inflation factors for all predictors across these nine models ranged from 1.00–1.16, indicating no meaningful multicollinearity.

### 3.3 Exploratory subgroup analyses (secondary)

Table 3 displays the results of the exploratory, ADI-stratified regression models, in which each social support subscale was entered independently after demographic covariates, stratified by dichotomized neighborhood disadvantage group and by race/ethnicity. As noted above, these subgroup comparisons are hypothesis-generating and do not themselves constitute a formal test of moderation (see Section 3.1).

**Table 3:** Exploratory, ADI-stratified regression models predicting global cognition (MMSE), by racial/ethnic group (secondary analysis).

Support subscale	*N*	R^2^ᶠᵘˡˡ	Model *F* (df)	B (SE)	*t*	*p*
Non-Hispanic Black — Low ND (ADI Quintiles 1–4)						
Demographics-only model (Model 1): R^2^ = 0.16–0.17, *F*(3, 643–646) = 40.38–45.18, *p* < 0.001						
Tangible	649	0.17	32.33*** (4,644)	0.278 (0.105)	2.65	*0.008*
Emotional	647	0.18	35.27*** (4,642)	0.215 (0.098)	2.18	*0.030*
Companion	650	0.16	30.48*** (4,645)	0.080 (0.098)	0.81	0.417
Non-Hispanic Black — High ND (ADI Quintile 5)						
Demographics-only model (Model 1): R^2^ = 0.19–0.19, *F*(3, 178–179) = 13.98–14.20, *p* < 0.001						
Tangible	182	0.19	10.52*** (4,177)	0.036 (0.181)	0.20	0.844
Emotional	183	0.19	10.46*** (4,178)	0.059 (0.174)	0.34	0.735
Companion	182	0.20	11.04*** (4,177)	0.226 (0.187)	1.21	0.229
Hispanic — Low ND (ADI Quintiles 1–4)						
Demographics-only model (Model 1): R^2^ = 0.35–0.36, *F*(3, 835–836) = 147.27–153.50, *p* < 0.001						
Tangible	840	0.35	114.28*** (4,835)	0.308 (0.096)	3.22	*0.001*
Emotional	839	0.36	117.07*** (4,834)	0.218 (0.094)	2.32	*0.021*
Companion	840	0.35	110.47*** (4,835)	0.057 (0.099)	0.58	0.565
Hispanic — High ND (ADI Quintile 5)						
Demographics-only model (Model 1): R^2^ = 0.21, *F*(3, 323) = 29.15, *p* < 0.001						
Tangible	327	0.22	22.46*** (4,322)	0.277 (0.191)	1.45	0.148
Emotional	327	0.21	21.85*** (4,322)	0.085 (0.196)	0.43	0.664
Companion	327	0.22	22.39*** (4,322)	0.276 (0.201)	1.37	0.171
Non-Hispanic White — Low ND (ADI Quintiles 1–4)						
Demographics-only model (Model 1): R^2^ = 0.08–0.08, *F*(3, 1,113–1,116) = 31.54–33.97, *p* < 0.001						
Tangible	1,118	0.09	28.98*** (4,1,113)	0.247 (0.069)	3.60	*< 0.001*
Emotional	1,120	0.08	24.42*** (4,1,115)	0.117 (0.069)	1.70	0.090
Companion	1,117	0.08	25.82*** (4,1,112)	0.082 (0.062)	1.32	0.188
Non-Hispanic White — High ND (ADI Quintile 5)						
Demographics-only model (Model 1): R^2^ = 0.16, *F*(3, 32) = 2.03, *p* = 0.129						
Tangible	36	0.19	1.81 (4,31)	0.306 (0.289)	1.06	0.298
Emotional	36	0.16	1.48 (4,31)	-0.003 (0.329)	−0.01	0.993
Companion	36	0.16	1.52 (4,31)	0.101 (0.252)	0.40	0.693

***p < 0.001.

#### 3.3.1 Non-Hispanic Black group

Among NHB participants residing in areas of low neighborhood disadvantage (ADI Quintiles 1–4), the demographics-only model predicting global cognition was significant [*F*(3,645) = 40.38, *p* < 0.001; R^2^ = 0.16], with age and education as the only significant predictors of MMSE performance. When each social support subscale was entered independently after these covariates, both Tangible Support [*F*(4,644) = 32.33, *p* < 0.001; R^2^ = 0.17; B = 0.278, SE = 0.105, *t* = 2.65, *p* = 0.008] and Emotional Support [*F*(4,642) = 35.27, *p* < 0.001; R^2^ = 0.18; B = 0.215, SE = 0.098, *t* = 2.18, *p* = 0.030] emerged as significant positive predictors of MMSE performance, whereas Companion Support did not [*F*(4,645) = 30.48, *p* < 0.001; R^2^ = 0.16; B = 0.080, SE = 0.098, *t* = 0.81, *p* = 0.417].

Among NHB participants residing in areas of high neighborhood disadvantage (ADI Quintile 5), the demographics-only model was also significant [*F*(3,178) = 14.09, *p* < 0.001; R^2^ = 0.19], with age and education again the only significant predictors. None of the three social support subscales significantly predicted MMSE performance in this stratum after accounting for demographics (Tangible: B = 0.036, SE = 0.181, *t* = 0.20, *p* = 0.844; Emotional: B = 0.059, SE = 0.174, *t* = 0.34, *p* = 0.735; Companion: B = 0.226, SE = 0.187, *t* = 1.21, *p* = 0.229), although each subscale’s inclusion still produced a significant overall model (Fs = 10.46–11.04, ps < 0.001; R^2^ = 0.19–0.20).

#### 3.3.2 Hispanic group

Among Hispanic participants residing in areas of lower neighborhood disadvantage (ADI Quintiles 1–4), the demographics-only model was significant [*F*(3,836) = 147.27, *p* < 0.001; R^2^ = 0.35], with age and education as significant predictors. Tangible Support [*F*(4,835) = 114.28, *p* < 0.001; R^2^ = 0.35; B = 0.308, SE = 0.096, *t* = 3.22, *p* = 0.001] and Emotional Support [*F*(4,834) = 117.07, *p* < 0.001; R^2^ = 0.36; B = 0.218, SE = 0.094, *t* = 2.32, *p* = 0.021] were each significant positive predictors of MMSE performance when entered independently, while Companion Support was not [*F*(4,835) = 110.47, *p* < 0.001; R^2^ = 0.35; B = 0.057, SE = 0.099, *t* = 0.58, *p* = 0.565].

Among Hispanic participants residing in areas of high neighborhood disadvantage (ADI Quintile 5), the demographics-only model was significant [*F*(3,323) = 29.15, *p* < 0.001; R^2^ = 0.21], with education as the only significant predictor. None of the three support subscales significantly predicted cognition in this stratum (Tangible: B = 0.277, SE = 0.191, *t* = 1.45, *p* = 0.148; Emotional: B = 0.085, SE = 0.196, *t* = 0.43, *p* = 0.664; Companion: B = 0.276, SE = 0.201, *t* = 1.37, *p* = 0.171), though each model remained significant overall (Fs = 21.85–22.46, ps < 0.001; R^2^ = 0.21–0.22).

#### 3.3.3 Non-Hispanic White group

Among NHW participants residing in areas of lower neighborhood disadvantage (ADI Quintiles 1–4), the demographics-only model was significant [*F*(3,1,114) = 33.97, *p* < 0.001; R^2^ = 0.08], with age, education, and sex as significant predictors. Tangible Support was a significant positive predictor of MMSE performance when added independently [*F*(4,1,113) = 28.98, *p* < 0.001; R^2^ = 0.09; B = 0.247, SE = 0.069, *t* = 3.60, *p* < 0.001], whereas neither Emotional Support [*F*(4,1,115) = 24.42, *p* < 0.001; R^2^ = 0.08; B = 0.117, SE = 0.069, *t* = 1.70, *p* = 0.090] nor Companion Support [*F*(4,1,112) = 25.82, *p* < 0.001; R^2^ = 0.08; B = 0.082, SE = 0.062, *t* = 1.32, *p* = 0.188] reached significance.

Among NHW participants residing in areas of high neighborhood disadvantage (ADI quintile 5), the demographics-only model was not significant [*F*(3,32)=2.03, *p* = 0.129; R^2^ = 0.16], and none of the three social support subscales was a significant predictor of MMSE performance in this small stratum (*n* = 36) (Tangible: B = 0.306, SE = 0.289, *t* = 1.06, *p* = 0.298; Emotional: B = -0.003, SE = 0.329, *t* = −0.01, *p* = 0.993; Companion: B = 0.101, SE = 0.252, *t* = 0.40, *p* = 0.693).

### 3.4 Sensitivity analyses

To evaluate the robustness of the primary findings reported above, we conducted four sensitivity analyses: (1) a diagnostic screen of the primary models, (2) a fully adjusted model accounting for cognitive diagnosis, depressive symptoms, and language of assessment, (3) a restriction to cognitively normal participants, and (4) re-estimation with ADI modeled as a continuous measure.

- MMSE outliers retained rather than excluded. All primary models reported in Table 1 retain the full analytic sample with valid MMSE data. To assess whether these estimates depended on a small number of overly influential cases, we screened each model using three standard diagnostics that flag cases with an extreme prediction error, an unusual combination of predictor values, or an outsized influence on the model’s estimated relationships (59), and re-estimated each model after excluding any case flagged by at least one criterion (6–10% of cases per model, depending on subscale and group). Seven of the nine associations were directionally and statistically consistent with the primary analysis after this exclusion: Hispanic Tangible Support (B = 0.269, *p* < 0.001), Hispanic Emotional Support (B = 0.242, *p* = 0.001), and White Tangible Support (B = 0.119, *p* = 0.011) all remained significant, and Companion Support remained non-significant in every group. Two associations shifted to non-significance: Black Tangible Support, significant in the primary analysis (B = 0.221, *p* = 0.015), was attenuated to non-significance (B = 0.129, SE = 0.072, *p* = 0.073), and Black Emotional Support, also significant in the primary analysis (B = 0.178, *p* = 0.037), was attenuated to non-significance (B = 0.025, SE = 0.073, *p* = 0.741). Taken together, these results suggest that associations observed among Black individuals are the least robust of the five primary associations to the influence of individual observations, and that any single finding should be interpreted alongside this sensitivity check rather than in isolation.
- Adjustment for cognitive diagnosis, depressive symptoms, and language of assessment. When consensus cognitive diagnosis, depressive symptom score, and language of assessment were added to each model, all nine associations attenuated substantially, and none remained statistically significant. Because diagnosis and depressive symptoms may lie within broader pathways linking social support and cognition, the attenuation observed after adjustment should not be interpreted as evidence of mediation. Rather, formal longitudinal and mediation analyses will be necessary to clarify these relationships.
- Restriction to cognitively normal participants. To address the possibility of reverse causation, we re-estimated all nine models restricted to participants with a consensus diagnosis of normal cognition (*n* = 523–933). Associations were attenuated and most no longer reached significance (all *p* ≥ 0.091), with the exception of White Tangible Support, which remained significant (B = 0.087, SE = 0.041, *p* = 0.032).
- ADI modeled as a continuous, quintile-based measure. Results were materially unchanged in direction, magnitude, and significance pattern across all nine models when ADI was modeled as a continuous quintile rank rather than dichotomized (e.g., Tangible Support: Black B = 0.217, *p* = 0.017; Hispanic B = 0.301, *p* < 0.001; White B = 0.251, *p* < 0.001; Emotional Support: Black B = 0.181, *p* = 0.034; Hispanic B = 0.185, *p* = 0.033). This indicates that our substantive conclusions do not depend on the decision to dichotomize ADI.

Considered together, these four sensitivity analyses point to a graded pattern of robustness rather than a uniform one. White Tangible Support was the only association that remained significant across the influence-diagnostics screen, the continuous-ADI re-estimation, and the restriction to cognitively normal participants, making it the most robust finding in this study. Hispanic Tangible Support and Hispanic Emotional Support were robust to the influence-diagnostics screen and to modeling ADI continuously but attenuated to non-significance when the sample was restricted to cognitively normal participants. Black Tangible Support and Black Emotional Support were the least robust of the five primary associations, both attenuating to non-significance under the influence-diagnostics screen alone. None of these patterns depended on how ADI was operationalized.

## 4 Discussion

This cross-sectional study investigated whether the association between social support and global cognitive functioning varied by level of neighborhood disadvantage in a diverse sample of adults and older adults with and without cognitive impairment. The formal interaction test did not provide statistical evidence that neighborhood disadvantage moderates this association in any racial/ethnic group. Independent of neighborhood disadvantage, Tangible Support was positively associated with cognition among Black, Hispanic, and White participants, and Emotional Support was positively associated with cognition among Black and Hispanic participants. Companion Support was not significantly associated with cognition in any group. In exploratory subgroup analyses stratified by neighborhood disadvantage, both Tangible and Emotional Support were significantly associated with cognition among NHB and Hispanic individuals residing outside of the most disadvantaged neighborhoods, and Tangible Support was also significant among NHW individuals in the same stratum. Significant associations were not observed within the smaller, most-disadvantaged subgroups.

Current findings align with extant literature suggesting that higher levels of social support are associated with improved cognition, underscoring the notion that social support confers a range of benefits to health and well-being (12, 28, 50). Given the cross-sectional design of this study, however, these findings should be understood as an association rather than evidence of a causal effect. Because the formal interaction test did not support moderation by neighborhood disadvantage, the exploratory pattern described above (associations concentrated among individuals living outside the most disadvantaged ADI quintile) reflects an asymmetric comparison between differing cell sizes and statistical power, and should not be read as evidence that neighborhood disadvantage moderates this relationship. Nevertheless, the exploratory subgroup pattern may be relevant to theories proposing that social support operates within broader environmental stress contexts, though the present study did not find statistical evidence of moderation by neighborhood disadvantage (51, 52).

Interestingly, positive associations between Tangible Support and cognition were only observed across Black, Hispanic, and White individuals in the pooled analysis, and Emotional Support was additionally associated with cognition among Black and Hispanic participants specifically. In the exploratory ADI-stratified subgroup comparisons, both Tangible and Emotional Support were significantly associated with cognition among Black and Hispanic individuals residing in neighborhoods of relative advantage. Although a variety of factors may contribute to this finding, it aligns with prior literature noting familial emotional support is particularly beneficial for cognition in Mexican adults as well as the joint, positive effects on memory outcomes associated with social support and education for African Americans (25, 53). This distinction parallels evidence that the cognitive relevance of Emotional Support, relative to other positive psychosocial factors, differs significantly across Black, Hispanic, and White older adults (54), underscoring that its salience for cognition may be shaped by ethnoracial context. Furthermore, Sims et al. (2011) showed that social support is positively associated with increased executive functioning in African American adults after controlling for demographic factors (50). Similar ethnoracial differences have been observed in young adults as well regarding the protective effect of social support on factors associated with cognition, such as anxiety and depression (55). Specifically, this relationship has been shown to be particularly important for Hispanic college students as compared to non-Hispanic White students (56). Among White participants, Tangible Support showed a significant positive association with cognition in the pooled analysis, the largest standardized effect among the three groups (β = 0.106), consistent with the broader literature on this association, which is often based on samples comprised primarily of White individuals (12, 57). This Tangible Support association was also observed within the low-disadvantage ADI-stratified subgroup (β = 0.10, *p* < 0.001), though not within the smaller high-disadvantage subgroup (*n* = 36), possibly reflecting reduced statistical power in that stratum.

Our hypothesis that Emotional Support would be associated with cognition (Hypothesis 2) was partially supported. Emotional Support was significantly associated with cognition among Black and Hispanic participants in the pooled analysis, though not among White participants, while Tangible Support showed the most consistent pattern of association across all three groups. These findings are not fully discrepant from prior studies suggesting that Emotional Support is the most important factor for cognition (12), rather, they suggest that Tangible Support may be the most broadly relevant form of social support for cognition across ethnoracial groups, while Emotional Support represents an additional correlate of cognition specifically among Black and Hispanic participants. An additional aspect that may help to explain this difference is that Tangible Support can encompass receiving greater assistance with completing everyday activities that support brain health, such as attendance at medical appointments or medication adherence (16, 51). However, it is important to note that these associations, given their cross-sectional nature, may also be bidirectional. In other words, social support may promote cognitive functioning, but intact cognition may likewise facilitate the ability to garner and maintain social support. Since Hispanic and NHB populations face a higher incidence and earlier onset of ADRDs, Tangible and Emotional Support may be especially vital for mitigating risk and promoting brain health within these at-risk groups. Further, a lack of Tangible support may itself be a chronic stressor, such that individuals with differing degrees of stress at baseline (e.g., ethnoracial inequalities associated with SES) have limited external resources (e.g., Tangible support) when additional stressors arise, regardless of neighborhood context.

Notably, significant associations between social support and cognition were not detected among individuals living in areas of greatest disadvantage. Individuals living in such neighborhoods reported significantly lower social support than those living in neighborhoods of less disadvantage, which aligns with other studies linking neighborhood factors and social cohesion (52). Furthermore, this difference raises the possibility that contextual factors may influence observed associations between social support and cognition, though this pattern was most evident for Tangible and Emotional support. Additionally, despite the availability of individual social support, one’s neighborhood context may hinder participation in activities that could facilitate additional social connections or diminish consistency of receiving social support (e.g., holding multiple jobs, reduced availability of transportation, lack of discretionary income, or fewer available safe space). This potential explanation is also supported by the broadly lower levels of social support reported by the high neighborhood disadvantage group in this study. Similarly, significantly lower scores observed on the cognitive screening measure in the high neighborhood disadvantage group compared to the low disadvantage group may indicate a lower baseline level of cognitive reserve that could further elevate the “threshold” needed to benefit from social support. In other words, this may reflect baseline reductions in global cognition that may stem from reductions in resources or cognitive reserve (e.g., fewer years of education) among individuals living in highly disadvantaged neighborhoods.

Importantly, the robustness of these findings was variable across sensitivity analyses. Associations attenuated substantially after adjustment for cognitive diagnosis, depressive symptoms, and language of assessment, and most were no longer significant when analyses were restricted to cognitively normal participants. White Tangible Support was the only association that remained significant across multiple sensitivity analyses. Accordingly, the present findings should be interpreted as preliminary and hypothesis-generating rather than definitive evidence of stable associations between social support and cognition across groups.

Taken together, these findings have important implications for clinical care from a cognitive perspective. Specifically, significant associations between social support and cognition were not observed in the highest-disadvantage neighborhoods, which may reflect the greater strain faced by individuals with fewer socioeconomic resources when providing or utilizing support (53, 54, 58). Given the heightened ADRD risk within Hispanic and non-Hispanic Black populations, these findings underscore the need for culturally-informed and tailored clinical care. In highly disadvantaged neighborhoods, broader structural barriers such as food insecurity and reduced healthcare access may also play important roles in cognitive health and warrant further investigation alongside social support factors. Conversely, in less disadvantaged areas, emphasizing “acts of service” or Tangible Support from family, as well as fostering Emotional Support networks, may offer a culturally resonant avenues for intervention that may be particularly relevant within collectivist communities and especially salient for Black and Hispanic individuals, given the significant association observed between Emotional Support and cognition in these groups. However, as we did not directly measure collectivism or cultural values in this study, this interpretation should be considered a hypothesis for future research. Future research is needed to examine interventions that leverage these specific social support structures to reduce the risk of cognitive decline and bolster neurological resilience.

### 4.1 Limitations

Despite several significant strengths including the overall sample size and ethnic/racial diversity of the sample, a few limitations are notable. First, the cross-sectional design of this study ultimately limited our ability to assess causation within the relationships of interest, including identified Tangible and Emotional support associations with cognition. Second, although findings from this study highlight implications for Black and Hispanic racial/ethnic groups, our ability to draw broader conclusions about White individuals living in highly disadvantaged neighborhoods is limited by statistical power related to sample size, as the unequal distribution of participants across ADI strata gave the stratified models somewhat unequal power to detect similarly sized effects across groups. Notably, results were consistent when ADI was modeled as a continuous, quintile-based measure, suggesting that this pattern was not simply an artifact of our dichotomization approach. Third, because participants with dementia were retained in the analytic sample, some observed associations between social support and cognition may be confounded by needs for functional support among individuals with greater cognitive impairment. Future work should examine whether these associations differ across the cognitive spectrum (CN, MCI, dementia). Fourth, the use of a cognitive screening measure to assess global cognition also limits generalizability to more comprehensive cognitive outcomes such as those obtained during a neuropsychological assessment. Fifth, the inherent subjectivity of self-reporting perceived social support may limit the objectivity of these findings and introduce measurement variation across respondents. Lastly, because we did not correct for multiple comparisons (e.g., Bonferroni or false discovery rate) across the nine main-effect models in Section 3.2, these associations are best interpreted as nominally significant. Additionally, the attenuation of most associations across sensitivity analyses, particularly after adjustment for clinical covariates and restriction to cognitively normal participants, suggests that several findings may not be robust across analytic conditions.

### 4.2 Future directions

Despite strides to incorporate diverse populations in human-subjects research, exploration of how contextual factors function within ethnoracial groups remains sparse regarding the association between social support and cognition. The community-based data collection approach used in the HABS-HD study addresses this gap by gathering psychological and cognitive data that reflects U. S. population diversity, offering direct clinical applications for a broader range of individuals. Future work that builds from this approach to determine whether clinical interventions based on social support can be tailored to specific cultural groups is therefore needed. Ultimately, randomized clinical trials to assess the utility of social support as an intervention and modifiable risk factor of cognitive decline will be useful, and longitudinal data collection will facilitate assessment of causality to further elucidate the efficacy of this approach. Additionally, this study was not designed to assess the mechanisms by which Tangible and Emotional Support are associated with global cognition, and these potential mediating factors therefore warrant future study to enhance clinical recommendations and care. Future research should examine whether enhancing social support in disadvantaged neighborhoods and incorporating culturally-tailored approaches are associated with improved cognitive outcomes. Finally, replication of findings in other diverse groups emphasizing collectivism, such as Asian cultures, is warranted to expand and validate how and whether social support may be more beneficial for cognition in different ethnoracial groups.

## 5 Conclusion

Taken together, these findings suggest that social support is associated with better cognition among Black, Hispanic, and White adults, though not uniformly and with variable robustness across sensitivity analyses. Tangible Support showed the most consistent associations with cognition across groups, while Emotional Support was associated with cognition specifically among Black and Hispanic participants. The formal interaction analyses did not support moderation by neighborhood disadvantage, although exploratory subgroup analyses found significant associations primarily among individuals residing outside the most disadvantaged neighborhoods. These findings may help guide future investigation of culturally tailored approaches emphasizing Tangible and Emotional Support, particularly among Black and Hispanic adults, though replication and longitudinal research are needed to clarify the role of neighborhood context and evaluate the stability of these associations.

## Data Availability

The datasets presented in this study can be found in online repositories. The names of the repository/repositories and accession number(s) can be found at: https://dss.niagads.org/cohorts/health-and-aging-brain-study-health-disparities-habs-hd/.

## Health and aging brain study (HABS-HD) study team

HABS-HD MPIs: Sid E O’Bryant, Kristine Yaffe, Arthur Toga, Robert Rissman, & Leigh Johnson; and the HABS-HD Investigators: Meredith Braskie, Kevin King, James R Hall, Melissa Petersen, Raymond Palmer, Robert Barber, Yonggang Shi, Fan Zhang, Rajesh Nandy, Roderick McColl, David Mason, Bradley Christian, Nicole Philips, Stephanie Large, Joe Lee, Badri Vardarajan, Monica Rivera Mindt, Amrita Cheema, Lisa Barnes, Mark Mapstone, Annie Cohen, Amy Kind, Ozioma Okonkwo, Raul Vintimilla, Zhengyang Zhou, Michael Donohue, Rema Raman, Matthew Borzage, Michelle Mielke, Beau Ances, Ganesh Babulal, Jorge Llibre-Guerra, Carl Hill and Rocky Vig.

## Appendix

### Social support questionnaire items

Belonging support

1. If I wanted to go on a trip for a day, I would have a hard time finding someone to go with me.
2. If I decide one afternoon that I would like to go to a movie that evening, I could easily find someone to go with me.
3. I do not often get invited to do things with others.
4. If I wanted to have lunch with someone, I could easily find someone to join me.

Emotional support

1. I feel that there is no one I can share my most private worries and fears with.
2. There is someone I can turn to for advice about handling problems with my family.
3. When I need suggestions on how to deal with a personal problem, I know someone I can turn to.
4. If a family crisis arose, it would be difficult to find someone who could give me good advice about how to handle it.

Tangible support

1. If I were sick, I could easily find someone to help me with my daily chores.
2. If I had to go out of town for a few weeks, it would be difficult to find someone who would look after my house or apartment (plants, pets garden etc.).
3. If I was stranded 10 miles from home, there is someone I could call who could come and get me.
4. If I needed some help in moving to a new house or apartment, I would have a hard time finding someone to help me.
